# NanoPack2: population-scale evaluation of long-read sequencing data

**DOI:** 10.1093/bioinformatics/btad311

**Published:** 2023-05-12

**Authors:** Wouter De Coster, Rosa Rademakers

**Affiliations:** Applied and Translational Neurogenomics, VIB Center for Molecular Neurology, VIB, Antwerp, Universiteitsplein 1, Antwerp 2610, Belgium; Applied and Translational Neurogenomics, Department of Biomedical Sciences, University of Antwerp, Antwerp, Universiteitsplein 1, Antwerp 2610, Belgium; Applied and Translational Neurogenomics, VIB Center for Molecular Neurology, VIB, Antwerp, Universiteitsplein 1, Antwerp 2610, Belgium; Applied and Translational Neurogenomics, Department of Biomedical Sciences, University of Antwerp, Antwerp, Universiteitsplein 1, Antwerp 2610, Belgium

## Abstract

**Summary:**

Increases in the cohort size in long-read sequencing projects necessitate more efficient software for quality assessment and processing of sequencing data from Oxford Nanopore Technologies and Pacific Biosciences. Here, we describe novel tools for summarizing experiments, filtering datasets, visualizing phased alignments results, and updates to the NanoPack software suite.

**Availability and implementation:**

The cramino, chopper, kyber, and phasius tools are written in Rust and available as executable binaries without requiring installation or managing dependencies. Binaries build on musl are available for broad compatibility. NanoPlot and NanoComp are written in Python3. Links to the separate tools and their documentation can be found at https://github.com/wdecoster/nanopack. All tools are compatible with Linux, Mac OS, and the MS Windows Subsystem for Linux and are released under the MIT license. The repositories include test data, and the tools are continuously tested using GitHub Actions and can be installed with the conda dependency manager.

## 1 Introduction

Long-read sequencing from Pacific Biosciences and Oxford Nanopore Technologies (ONT) has evolved from single genomes and small groups of individuals to large population-scale cohorts (Beyter et al. 2021; De Coster et al. 2021). Simultaneously, the increasing economic cost and climate impact of computational tasks also necessitate more efficient bioinformatic methods for data quality assessment and processing (Pereira et al. 2017). However, several tools have been developed for the quality assessment of long-read sequencing data without scaling to populations of >100 genomes (Watson et al. 2015; De Coster et al. 2018; Lanfear et al. 2019; Leger et al. 2020). This article presents newly developed tools that fulfill this need and efficiently assess characteristics relevant to long-read genome sequencing, including alignments spanning structural variants and phasing read alignments. Phasing, i.e. assigning each sequenced fragment to a parental haplotype by identifying co-occurring variants (Martin et al. 2016; Edge and Bansal 2019), is critical in identifying potential functional variants in association studies and for the pathogenicity of putative compound heterozygous variation. Furthermore, we present an update on NanoPlot and NanoComp from the NanoPack tools (De Coster et al. 2018).

## 2 Software description

Improvements to NanoPlot and NanoComp are, among code optimizations, the generation of additional plots, using dynamic HTML plots from the Plotly library, and enabling further exploration by the end users (Supplementary Fig. S1). The tools now also support input using the programming language agnostic Arrow data format. A binary is provided to efficiently generate Arrow files from BAM/CRAM alignments. Chopper is a tool that combines the utility of NanoFilt and NanoLyse, for filtering sequencing reads based on quality, length, and contaminating sequences, delivers a 7-fold speed up compared to the Python implementation, making use of the Rust-Bio library (Köster 2016) and Rust bindings to minimap2 (Li 2018).

Summarization of long-read sequencing experiments using NanoStat (De Coster et al. 2018) is too slow considering the yields that are nowadays common with nanopore sequencing. Cramino, using rust-htslib (Köster 2016; Bonfield et al. 2021), provides a much faster alternative for gathering metrics based on the data output, mean coverage, the number of reads, their mean and median length, and sequence identity relative to the reference genome. Long reads span structural variants, and penalizing the read accuracy for a large gap is undesirable. For this reason, Cramino calculates the gap-compressed identity, defined as the edit distance relative to the read length, while counting consecutive alignment gaps as just one difference (Supplementary Methods). Cramino allows filtering on read length and optionally outputs a rudimentary evaluation of the karyotype and biological sex by calculating normalized read counts per chromosome, calculates the MD5 checksum to control for data integrity, provides metrics of the read phasing performance, and can provide metrics on the number of spliced exons for long-read transcriptomics. Importantly, Cramino remains compatible with the rich visualizations from NanoPlot and NanoComp by generating output in the Arrow format on top of the optional lightweight histograms for read length and read identity from Cramino itself. For ONT human genome sequencing with 50× coverage using four cores for BAM/CRAM decompression, Cramino takes 12 min with a peak memory usage of 147 Mb without optional output or 21 min with a peak memory usage of 690 Mb for total output including histograms, karyotype, phasing metrics, MD5 checksum, and generation of the Arrow file. Kyber is a tool for creating standardized plots of log-transformed read length versus (phred-scaled) gap-compressed reference identity, for a single input file or a comparison of up to three CRAM or BAM files (Supplementary Methods and Supplementary Fig. S2).

**Figure 1.**
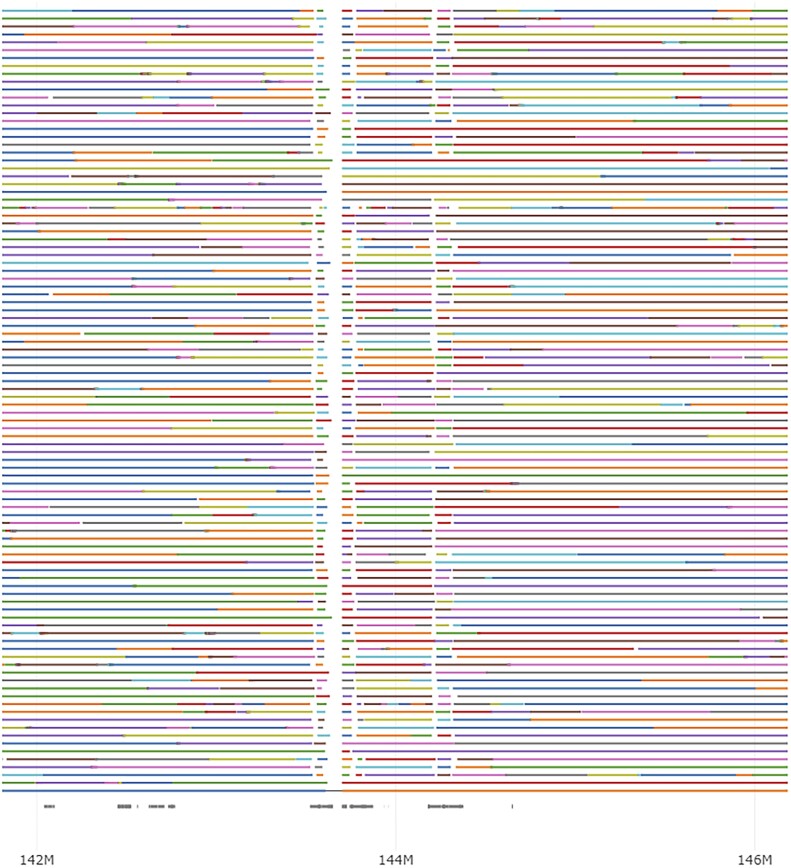
Example of phasius output. This plot shows the haplotype phasing structure of chr7:142 000 000–146 000 000 for 92 individuals. Every horizontal line is from a single individual, with a change in color indicating the start of a new contiguously phased genomic segment. The annotation track (bottom) shows segmental duplications with grey bars, predictably breaking the phased blocks in the case of longer repetitive elements. An interactive example can be found at https://wdecoster.github.io/phasius.

Phasius is developed to visualize the results of read phasing, which shows in a dynamic genome browser style the length and interruptions between contiguously phased blocks from a large number of individuals together with genome annotation, for example segmental duplications (Supplementary Methods and Fig. 1) (Bailey et al. 2002). Phasius takes 26 s to generate the example figure for 92 individuals in a 10 megabase interval, with eight parallel threads and a peak memory usage of 4.3Gbyte. For the example figure, reads were aligned with minimap2 (Li 2018), and alignments phased with longshot (Edge and Bansal 2019).

## 3 Conclusion

NanoPack now offers tools for evaluating large populations with implementations in a more performant programming language, focusing on features relevant to long-read sequencing. The software suite remains easy to install on all major operating systems and offers interactive visualization in HTML format.

## Supplementary Material

btad311_Supplementary_Data

## References

[btad311-B1] Bailey JA , Gu Z, Clark RA et al Recent segmental duplications in the human genome. Science 2002;297:1003–7. 12169732 10.1126/science.1072047

[btad311-B2] Beyter D , Ingimundardottir H, Oddsson A et al Long-read sequencing of 3,622 icelanders provides insight into the role of structural variants in human diseases and other traits. Nat Genet 2021;53:779–86. 33972781 10.1038/s41588-021-00865-4

[btad311-B3] Bonfield JK , Marshall J, Danecek P et al HTSlib: C library for reading/writing high-throughput sequencing data. Gigascience 2021;10:1–6. 10.1093/gigascience/giab007 PMC7931820 33594436

[btad311-B4] De Coster W , D'Hert S, Schultz DT et al NanoPack: visualizing and processing long-read sequencing data. Bioinformatics 2018;34:2666–9. 29547981 10.1093/bioinformatics/bty149 PMC6061794

[btad311-B5] De Coster W , Weissensteiner MH, Sedlazeck FJ et al Towards population-scale long-read sequencing. Nat Rev Genet 2021;22:572–87. 34050336 10.1038/s41576-021-00367-3 PMC8161719

[btad311-B6] Edge P , Bansal V. Longshot enables accurate variant calling in diploid genomes from single-molecule long read sequencing. Nat Commun 2019;10:4660. 31604920 10.1038/s41467-019-12493-y PMC6788989

[btad311-B7] Köster J. Rust-Bio: a fast and safe bioinformatics library. Bioinformatics 2016;32:444–6. 26446134 10.1093/bioinformatics/btv573

[btad311-B8] Lanfear R , Schalamun M, Kainer D et al MinIONQC: fast and simple quality control for MinION sequencing data. Bioinformatics 2019;35:523–5. 30052755 10.1093/bioinformatics/bty654 PMC6361240

[btad311-B9] Leger A , Leonardi T. a-slide/pycoQC: v2.5.0.23, 2020.

[btad311-B10] Li H. Minimap2: pairwise alignment for nucleotide sequences. Bioinformatics 2018;34:3094–100. 29750242 10.1093/bioinformatics/bty191 PMC6137996

[btad311-B11] Martin M et al WhatsHap: fast and accurate read-based phasing. bioRxiv, 085050, 2016. 10.1101/085050.

[btad311-B12] Pereira R et al Energy efficiency across programming languages: how do energy, time, and memory relate? In: *Proceedings of the 10th ACM SIGPLAN International Conference on Software Language Engineering, SLE 2017*, pp. 256–267. New York, NY, USA: Association for Computing Machinery, 2017.

[btad311-B13] Watson M , Thomson M, Risse J et al poRe: an R package for the visualization and analysis of nanopore sequencing data. Bioinformatics 2015;31:114–5. 25173419 10.1093/bioinformatics/btu590 PMC4271141

