# Supplementary material for: NanoPack2: population-scale evaluation of long-read sequencing data
Source: Bioinformatics. 2023 May 12;39(5):btad311. doi: 10.1093/bioinformatics/btad311 (PMC10196664; doi:10.1093/bioinformatics/btad311)
Supplement: btad311_Supplementary_Data [file btad311_supplementary_data.zip › NanoPack2-supplementary.docx]

**NanoPack2: Population scale evaluation of long-read sequencing data: Supplementary materials**

**Supplementary methods**

**Gap-compressed reference identity**

The gap-compressed reference identity, defined as the edit distance relative to the read length, while counting consecutive gaps as just one difference, as used by cramino and kyber, is preferentially obtained from the BAM *de* tag, as available from recent versions of the minimap2 aligner. For older versions of minimap2, or other aligners, the gap-compressed reference identity is calculated per sequenced read from the BAM *NM* tag (standard edit distance) by subtracting the total size of insertions and deletions and the addition of the number of gap opens.

$$1.0-\frac{NM -gap_{size}+gap_{count}}{matches+gap_{count}}$$

**Standardized raster and transformations in kyber**

Kyber uses a 600-by-600 raster to show a 2D heatmap of reads based on their log_10_-transformed read-length and gap-compressed reference identity, either in percentages or Phred-scaled. Read lengths are capped to 1 megabase or 6 on the log_10_ scale. The x-axis space is further divided in 100 bins per order of magnitude. On the y-axis, the data is capped at minimal 70% accuracy or maximal Q40 on the Phred scale, with the remaining space scaled to 600 values between respectively 70 and 100% or Q0 and Q40. After transforming the input reads in the 600 read length and 600 accuracy bins, internally stored using a HashMap, the highest scoring bin will obtain the most intense color (e.g. RGB (0, 0, 255)), and all bins with fewer reads are scaled relatively. When comparing two datasets, a common maximum value is used for normalization, and cells overlapping between both datasets, two or three color channels are used to show the relative intensities (e.g. RGB (123, 0, 60), with dataset 1 using the red channel and dataset2 the blue channel, reaching various shades of purple).

**Alignment blocks in phasius**

Per CRAM/BAM input file, phasius will iterate over all aligned reads in the selected interval to extract phased reads and their start and end coordinates while excluding secondary alignments. Phase blocks are then constructed by iterating over the collected phased reads and extending the block until a new phase set identifier (BAM PS tag) is encountered. Annotation is added to the plot extracted from a BED file for all intervals that overlap the selected region based on a tabix index.

**Figure S1**

| **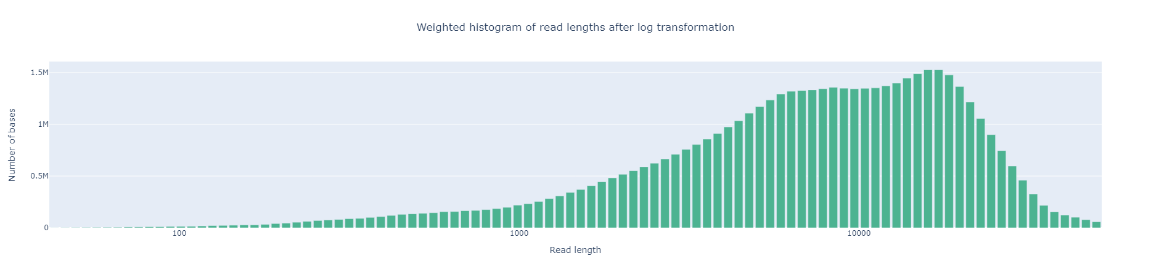** |
| --- |
| **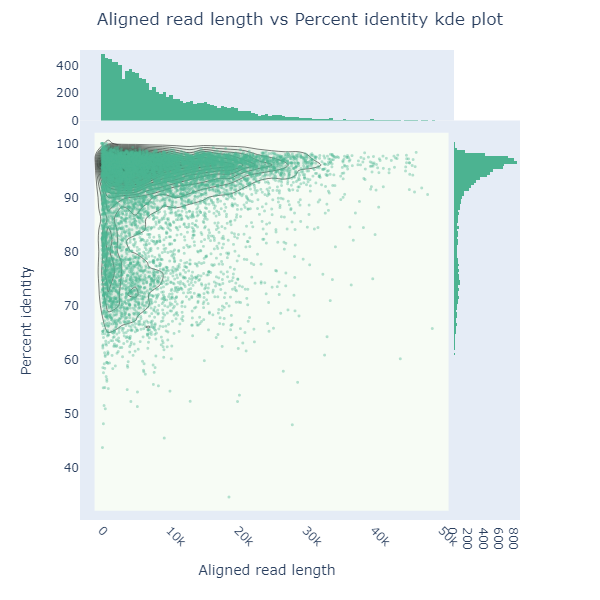** |
| **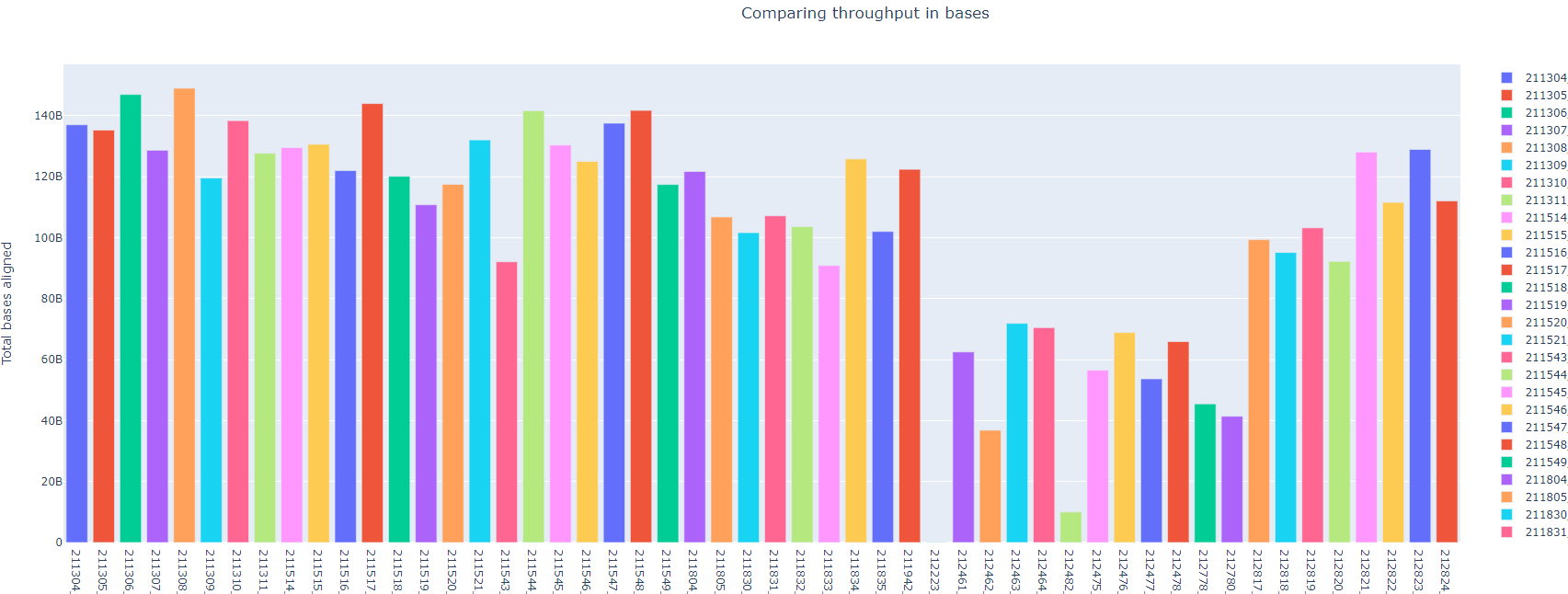** |
| Example figures of NanoPlot and NanoComp:  top: log-transformed read length histogram weighted by number of bases per bin (NanoPlot) middle: scatter plot of aligned read length and read percent identity (NanoPlot)  bottom: comparison of sequencing data across runs (NanoComp) |

**Figure S2**


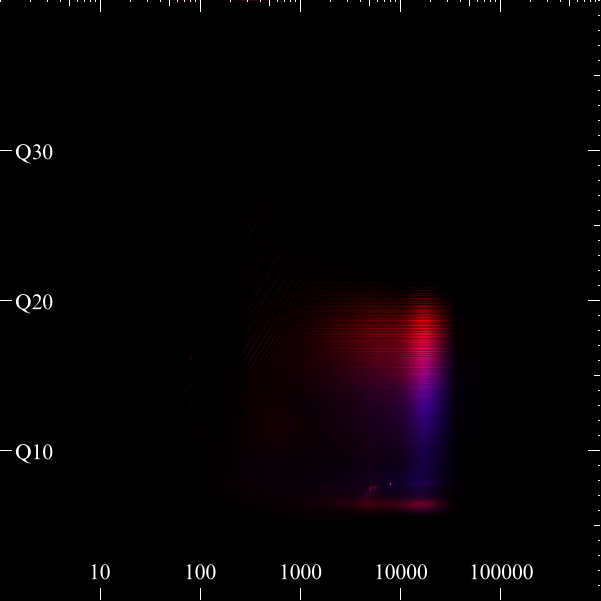


Example figure of kyber output. The plot shows the log-transformed read length on the x-axis and the phred-scaled gap compressed reference identity on the y-axis. Both axes are standardized and always use the same scale for easy comparison. An ONT PromethION library from a human genome sequencing on an R9.4.1 flow cell and LSK109 library preparation is shown in blue, and data from an R10.4.1 flow cell and LSK114 library in red, with both libraries generated from the same DNA sample. Overlapping locations are shown in shades of purple.
