# Supplementary figures and images for: NanoPack2: population-scale evaluation of long-read sequencing data
Source: Bioinformatics. 2023 May 12;39(5):btad311. doi: 10.1093/bioinformatics/btad311 (PMC10196664; doi:10.1093/bioinformatics/btad311)

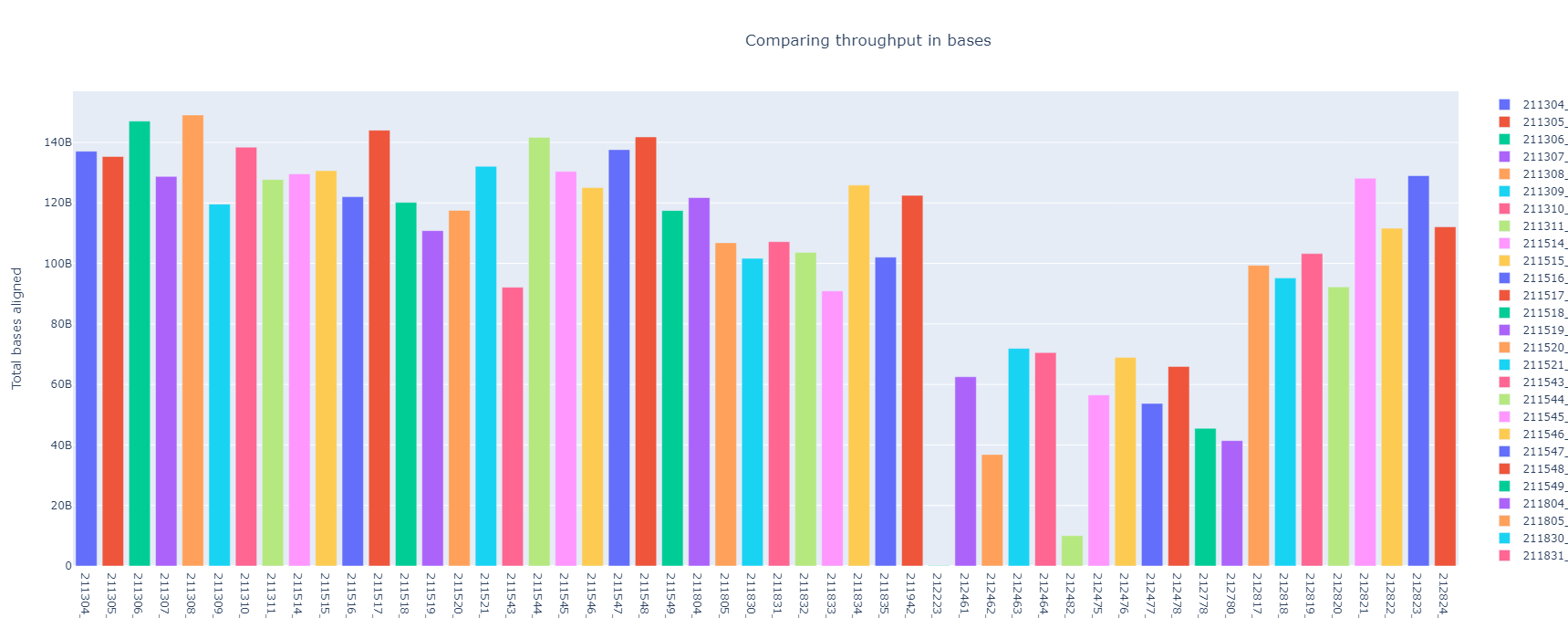

Supplement: btad311_Supplementary_Data [file btad311_supplementary_data.zip › FigureS1_C.png]

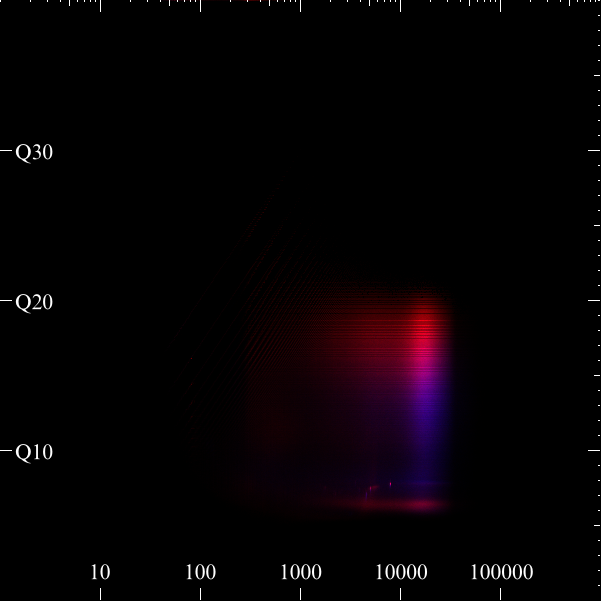

Supplement: btad311_Supplementary_Data [file btad311_supplementary_data.zip › FigureS2.png]

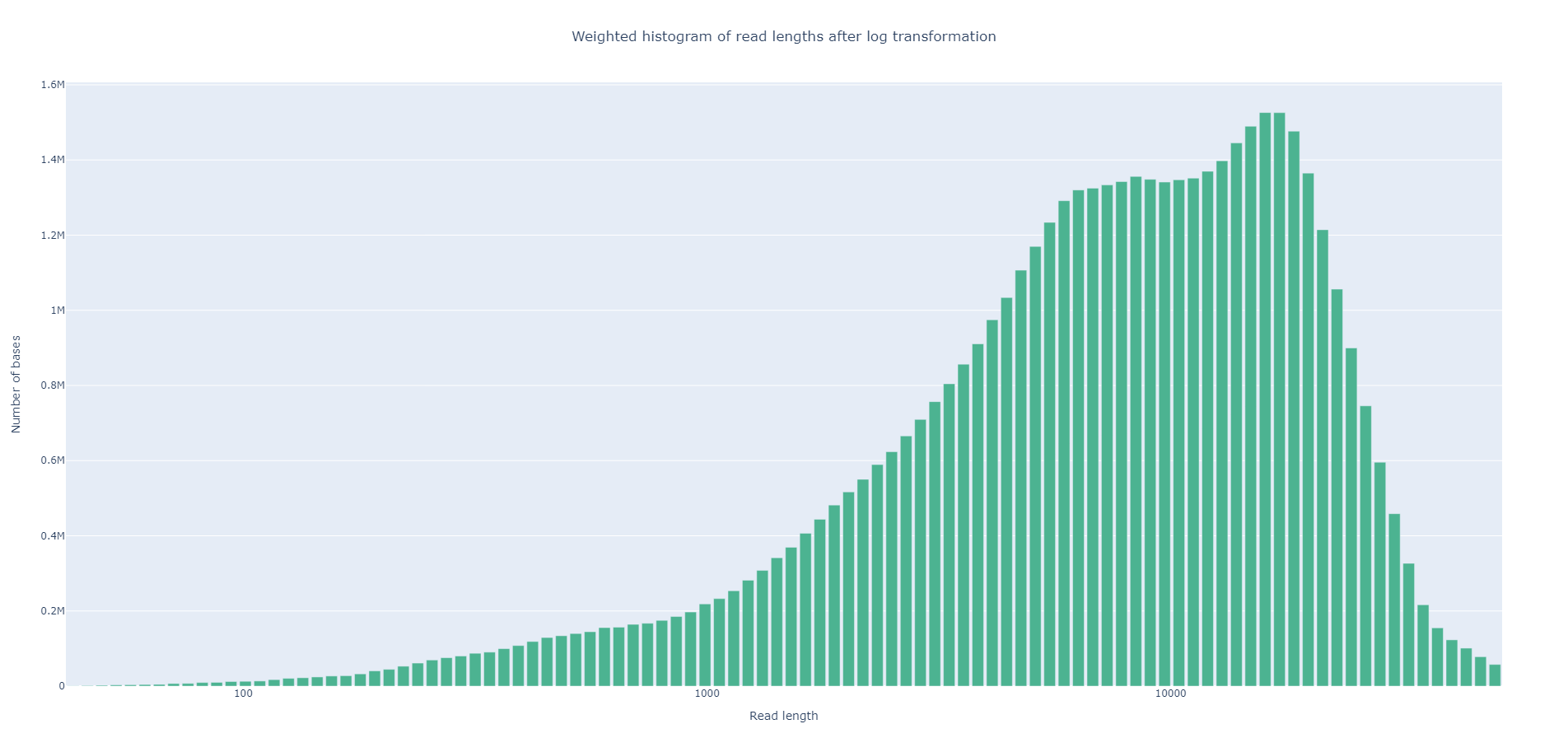

Supplement: btad311_Supplementary_Data [file btad311_supplementary_data.zip › FigureS1_A.png]

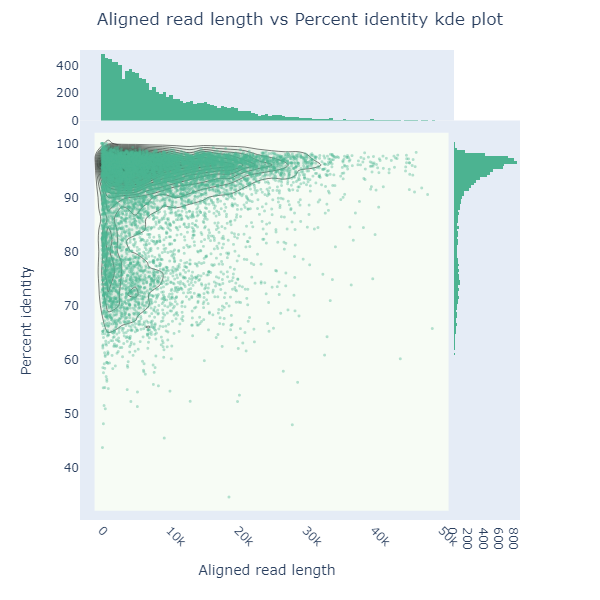

Supplement: btad311_Supplementary_Data [file btad311_supplementary_data.zip › FigureS1_B.png]
